# Identifying challenges to critical incident decision-making through a macro-, meso-, and micro- lens: A systematic synthesis and holistic narrative analysis

**DOI:** 10.3389/fpsyg.2023.1100274

**Published:** 2023-03-28

**Authors:** Brandon May, Rebecca Milne, Andrea Shawyer, Amy Meenaghan, Eva Ribbers, Gary Dalton

**Affiliations:** School of Criminology and Criminal Justice, University of Portsmouth, Portsmouth, United Kingdom

**Keywords:** critical incident, emergency response, narrative analysis, decision making, cognition, bibliometric analysis, systematic synthesis

## Abstract

It is predicted that emergency responses to critical incidents will increase over the next few decades, as society faces unique and dynamic challenges (e.g., pandemics, migrant crises, and terrorism). As such, it is necessary to breakdown, identify, and evaluate the unique barriers associated with decision-making in the context of critical incident responses. The aim of the current study was to synthesize the bibliographic characteristics of the research on decision making and present a holistic narrative analysis of the multi-layered factors. Additionally, the systematic synthesis of evidence facilitated a critical appraisal of the quality and distribution of evidence across macro-, meso-, and micro- levels. Results suggested that research was moderately heterogeneous, as evidence captured diverse narrative factors. However, micro-centric characteristics (e.g., cognitive-related factors) were not well represented. Instead, research primarily focused toward intermediate meso-level characteristics, capturing factors such as “*interoperability*” and “*organization policy and procedure*” as critical challenges to decision-making. Six key narratives were also identified and discussed. Both the quality appraisal and narrative findings suggested that research should seek opportunities to experimentally assess, evaluate and validate decision-making. Whilst this has previously appeared ethically and practically problematic, advances in technology, research and analysis have allowed high-fidelity simulation experimentation to recreate critical incidents.

## Introduction

Over the next few decades, it is predicted that emergency responses to critical incident events—often characterized by their complexity, uncertainty, high-risk, and high-stakes (Power and Alison, 2017)—will significantly increase. This increase will require large-scale deployments of multi-agency systems in the response and recovery of an incident (Timperio et al., 2016). For example, there has been a steady increase in mass causality events (e.g., terror related incidents; Craigie et al., 2020) that require a rapid and actionable response from individual agencies (e.g., Police; Fire and Rescue; and Critical Care operators). Further, these emergency response agencies are often required to operate within the boundaries of a multi-agency system (Brown et al., 2020). Operating within these contexts often extends operational capabilities of individual response agencies and multi-agency systems beyond their *normal* scope of duty (House et al., 2014), limiting their operational effectiveness and impeding effective operational, tactical, and strategic decision-making (Alison et al., 2018; Shortland et al., 2020).

In a broader context, current challenges faced by emergency responders, such as the response to COVID-19 (Stevens, 2020; Ghaemmaghami et al., 2021; Newiss et al., 2021; Stanier and Nunan, 2021) and right wing and domestic terrorism (e.g., Smith and Barrett, 2019; Hayes, 2021), have continued to stretch operational capabilities beyond operational policy, practice, and procedure (House et al., 2014; Cohen-Hatton et al., 2015; Power and Alison, 2017; Alison et al., 2018). For example, key decision-makers *in-situ* of critical incidents have often struggled to commit to *choice* when presented with competing and often unreliable information (Power and Alison, 2017; Smith and Milne, 2018). In part, this may be due to standard operating procedures and emergency management policy failing to provide clarity toward ethical critical incident decision-making (e.g., Kapucu and Garayev, 2011; Rebera and Rafalowski, 2014). However, research has yet to fully explore this. In addition, operational procedures, and frameworks (e.g., Civil Contingency Act, 2004; Joint Decision Model, 2022) developed to enhance critical incident decision-making, have often lacked contextual discourse to situational specific responses, as scientific evidence used to inform policy, legislation and evidence-based frameworks have lacked objectivity (Stevens, 2020). That is, the systematic and iterative processes that serve to build confidence in, or identify flaws in the operational procedural approach, are yet to fully capture the nuance of scientific evidence in a non-biased format (Stevens, 2007, 2020). For instance, research has demonstrated that current models of the policy-evidence relationships have often neglected to focus on the breadth of evidence, instead focusing on the interests that best suit powerful social agendas (Stevens, 2007, 2020). Research has also highlighted that current critical incident response systems have lacked triaging systems to support critical incident decision-making in respect to witness trauma (Smith and Milne, 2018), and key decision-makers have lacked transferable experience toward critical incident decision-making (see Alison et al., 2015b) resulting in reduced situational awareness (Power and Alison, 2017).

There has been a continued effort toward enhancing the understanding of decision-making factors that challenge effective response (e.g., House et al., 2014; Shortland et al., 2020). For example, understanding the challenges that exist in response to an Islamic extremist terror attack (e.g., Manchester Arena Bombing), an international response to a pandemic resulting in national-level public lockdowns (e.g., COVID-19; Stevens, 2020), or a national response to a growing migrant crisis (e.g., the UK migrant crisis; Home Office, 2022). However, there has been a sparsity of research that has sought to synthesize these challenges. More specifically, research that seeks to synthesize the challenges through a multi-dimensional lens that brings together and highlights the implications on practitioners and policy makers to mitigate and create reactive, flexible strategy, policy, and protocols (Power and Alison, 2017). Gil-Garcia et al. (2016) presented such an approach, highlighting operational challenges at a sociological macro-level (e.g., politically driven factors). They found that detachment from political discourse, policy and emergency management doctrine that identified response hierarchy and leadership, was not possible. Thus, influencing effective critical incident decision-making in response to the World Trade Center attacks in 2001. Yet, this study only presented a single sociologically driven macro perspective. It is widely acknowledged that decision-making can also be driven by sociologically and psychologically driven macro- (e.g., politically driven factors; Kapucu, 2009; Stevens, 2020), meso- (e.g., organizationally driven factors; Alison et al., 2015a), and micro- (e.g., cognitive-related factors; Power and Alison, 2017) factors. Thus, the need for a more compressive study that seeks to examine these factors.

Given this, it is important to identify and clarify the unique factors that exist at critical incident events by representing and synthesizing decision-making barriers through a multi-layered dimensional lens. Previous synthesis of decision-making research (e.g., Alison et al., 2013) has focused on the unique barriers of decision-making, and this has been shown to offer critical insight embodied in multi-agency systems and individual response agencies (Wilkinson et al., 2019). This paper aimed to explore and identify the holistic factors of decision-making challenges through a multi-dimensional lens by undertaking a systematic synthesis of evidence. Further, challenges were narratively evaluated through a holistic narrative analysis—a technique that allows analysis and observation of real-world practices across a wide range of interactions (e.g., Davidson et al., 2005).

## Method

**Figure 1:**
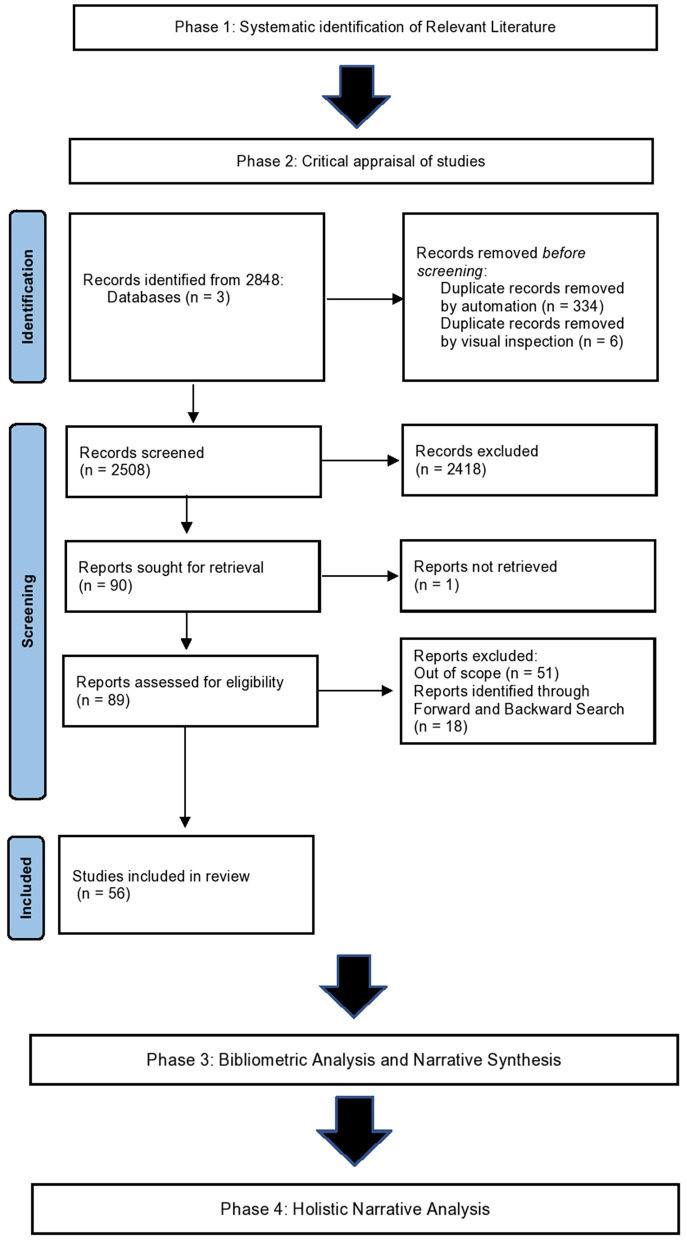
PRISMA criteria described by Page et al. (2021). The PRISMA 2020 statement: an updated guideline for reporting systematic reviews.

This study presents a systematic synthesis of the literature, and identifies key narratives associated with decision-making by coding macro-, meso-, and micro- levels. Data was collected from publicly available databases; as such, no formal recruitment of participants was required. Approval for the study was granted by the University of Portsmouth Ethics Committee, where it was deemed exempt from further ethical review due to the use of secondary datasets and synthesis of extant materials. In seeking to understand the challenges of decision-making in critical incidents, the current study comprised of four distinct phases—an accepted and adapted method utilized by House et al. (2014): (1) the systematic identification of relevant literature, policy, and policy-related documents; (2) a critical appraisal of all eligible documents; (3) a bibliometric analysis and narrative synthesis; and (4) the narrative coding of the data, to present primary narrative themes (see Figure 1 for the full PRISMA framework breakdown). It is worth noting, that whilst it is recognized that some literature may be obtainable through non-traditional channels (e.g., gray literature), this paper did not seek out such literature.

### Phase 1: Systematic identification of relevant literature

Data retrieval commenced in July 2021 and ceased in July 2022. All sources were obtained using the library tools “EBSCO Discovery” and “Web of Science”, which search a number of databases representing over 3.7 billion records from academic publishers and information providers (e.g., PsycINFO, JSTOR, Wiley, and Taylor and Francis Online; see *EBSCO Discovery Service* and *Web of Science* for a more detailed overview). In addition, materials were obtained *via* a Google Scholar search. All policy and legislative documents were obtained from GOV.uk.

The search strategy focused primarily on a highly sensitive approach, rather than a highly specific approach, allowing for the identification of literature relevant to the research question (see Table 1, for an extract of these terms). For example, terms such as “Critical Incident” AND “Decision-Making” or “Emergency Response” OR “Critical Incident” AND “Interoperability” were used to help identify relevant literature. Such an approach ensured results captured an exhaustive list of the possible factors that influence decision-making in emergency response. Given the sparsity of research specifically related to critical incident events, the search criteria extended the literature search to capture research related to “*critical incidents*”, “*major incidents*” and “*other emergency response”* scenarios. Published peer-reviewed papers were included if, (i) they were written in English, (ii) had a primary focus toward emergency response decision-making, and (iii) captured experiential, experimental, or case study data across individual response domains and/or multi-agency systems. Policy and legislative documents were also reviewed, and were included if they provided direct guidance, policy, procedure, or processes in emergency response. Studies were excluded if they did not relate to emergency response. No time frame was specified for the search, and so all relevant papers identified in the review before December 2021 were included.

**Table 1:** An extract of the key terms used to identify relevant literature.

**Key terms**	**Alternative examples**
Decision-making	Situational awareness, interoperability, common ground
Response	Crisis response, emergency response, emergency management
Macro-, meso-, and micro-	Cognitive, organizational, social, political
Incident type	Accident, disaster, terrorism
Case studies	9/11, Manchester arena, Grenfell, COVID-19

### Phase 2: Critical appraisal of the literature

The primary author undertook an initial scoping review, which yielded 2,508 articles for potential inclusion, after all duplicate reports (*N* = 340) were removed. After title and abstract screening, 2,418 papers were removed from the analysis for not meeting the inclusion criteria. Of the 90 published articles remaining, one article was not publicly accessible and was removed, which left 89 articles, all of which underwent full text screening. Subsequently, 51 of the 89 articles were removed for not meeting the inclusion criteria; for example, articles which represented critical incident decision-making in acute hospital settings were considered to lack relevance to frontline emergency response. An additional 18 articles were identified *via* forward and backward searching. This left 56 published research articles for analysis.

All articles were electronically available, and an initial coding framework was developed to capture the primary narratives before these were then categorized by their macro-, meso-, and micro- characteristics (see Table 2, for the definition of the macro-, meso- and micro- components used to categorize eligible articles). Note, this paper sought to identify key narratives by identifying first order (i.e., the primary focus of the assessed paper) and sub-level (e.g., whether narratives focused on meso-level factors, such as interoperability) narratives, and did not seek to identify peripheral narratives (e.g., secondary research aims), which may have been identifiable through a more fine-grained narrative analysis.

**Table 2:** Definition of the macro-, meso-, and micro- components used to categorize eligible articles. Definitions have been adapted from Smith and Barrett (2019).

**Components**	**Definition**	**Examples**
Macro	Perceived political, legal, regulatory, ethical, and/or economic external conditions that are considered beyond the influence of (i) an organization, or (ii) an individual	Inadequacy of funding; politically driven regulatory systems
Meso	Perceived local organizational factors that influence operational parameters and/or service delivery.	Interoperability; organizational systems and policy
Micro	Perceived factors and/or characteristics that are considered to influence decision-making at an individual level.	Cognitive inertia (e.g., decision-inertia); aversion to personal risk

### Phase 3: Bibliometric and narrative synthesis

Narrative synthesis has been shown to consistently provide a nuanced and comprehensive insight into complex multi-agency emergency response decision-making (e.g., House et al., 2014; Penney et al., 2022). Eligible articles were initially coded independently by the principal investigator, allowing for the distribution of research to be categorized by macro-, meso-, and micro- levels (e.g., whether articles focused primarily on cognitive factors, organizational factors, or political factors). Based on well-established methods for coding, indexing, and generating narrative links between data (see, Corbin and Strauss, 2015), an additional independent researcher coded all of the data for first order narratives, with consideration given to where articles presented multiple first order narratives, and sub-level narratives based on the pre-defined categories of what constitutes macro-level evidence (e.g., political-centric challenges), meso-level evidence (e.g., organizational challenges), and micro-level evidence (e.g., cognitive challenges). All researchers agreed on the narratives assigned to each paper.

**Figure 2:**
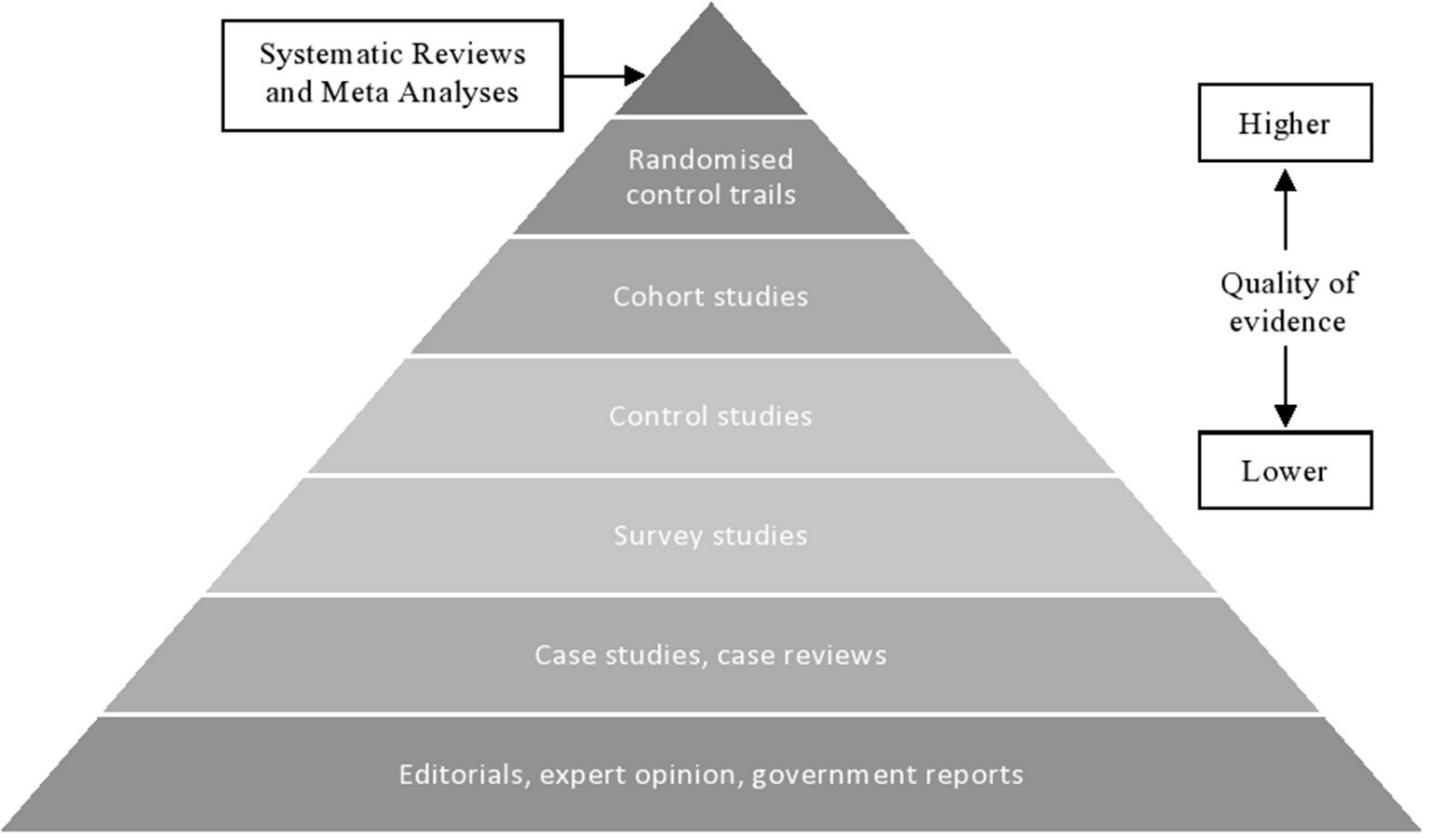
The evidence of hierarchy pyramid. See, Brighton et al. (2003).

In an attempt to critically appraise the quality of evidence, and in accordance with best practice (Petticrew et al., 2013), a bibliometric quality assessment was undertaken. However, there was little consensus within the research team of how best to conduct a reliable and robust quality assessment. As such, the principal investigator relied on coding eligible evidence based on the *Hierarchy of Evidence (HoE)* Framework (see Figure 2). Of the eligible studies, the principal investigator identified that research was often considered as being middling or low quality (i.e., research evidence seldom adopted a research methodology beyond case reviews; *N* = 35). Five studies were identified as having a very high-quality position on the HoE (i.e., systematic reviews and case syntheses) and sixteen studies adopted an experimental epistemology. Twenty percent of all eligible studies were independently coded, using the same HoE framework. Inter-rater reliability assessment revealed that there was 100% agreement between the coders.

### Phase 4: Holistic narrative analysis

The primary researcher undertook a modified narrative analysis—holistic narrative analysis by independently coding sub-level and primary narratives of secondary data sources (i.e., published literature; Popay et al., 2006). The primary function of this was to present common elements that theorized and conceptualized challenges to decision making across and within the emergency response literature. This allowed for the presentation of critical perspectives on complex factors by presenting narrative themes (e.g., see Riessman, 2008; McGannon and Smith, 2015). Further, this narrative inquiry shared similar features to thematic analysis (Braun and Clarke, 2006), as each approach attended to content of text (e.g., what is said) whilst maintaining a sense of interpretation in how the data was generated. As such, the principles of holistic narrative analysis allowed for broad interpretations of decision-making by identifying key narrative themes within the data to best describe the evidence.

The holistic narrative analysis consisted of coding for first-order narratives that could be used to best describe the papers (e.g., macro-, meso-, and micro- characteristics). Emergent narratives were then categorized into their respective sub-levels (see Table 3, for a detailed breakdown of these sub-levels). Further, a deductive, theory driven approach was adopted to deconstruct pre-existing decision-making theory in context to sub-level characteristics associated to critical incident and emergency response.

**Table 3:** Narrative bibliometric coding, showing the identification of first-order narratives categorized against sub-level narratives.

**Sub-level narratives**	**First-order narratives (%)**		
	**Primary**	**Secondary**	**Tertiary**
**Macro-centric**			
Politics, policy, and reform	13 (23.2)	2 (3.6)	0 (0.0)
Emergency management doctrine	**6 (10.7)**	**2(3.6)**	**0 (0.0)**
Ethical issues	**7(12.5)**	**0 (0.0)**	**0 (0)**
Economic issues	**1(1.8)**	**0 (0.0)**	**1 (1.8)**
Total	**27 (48.2)**	**4 (7.1)**	**1 (1.8)**
**Meso-centric**			
Interoperability, communication and coordination	16 (28.6)	4 (7.0)	0 (0.0)
Technology	**1 (1.8)**	**0 (0.0)**	**0 (0.0)**
Policy, procedure and process	**0 (0.0)**	**2 (3.6)**	**0 (0.0)**
Organizational structure	**1(1.8)**	**0 (0.0)**	**0 (0.0)**
Risk, uncertainty and avoidance	**4 (7.1)**	**1 (1.8)**	**0 (0.0)**
Total	**22 (39.3)**	**7 (12.5)**	**0 (0.0)**
**Micro-centric**			
Decision inertia	**3 (5.6)**	**3 (5.6)**	**0 (0.0)**
Risk aversion	**0 (0.0)**	**0 (0.0)**	**0 (0.0)**
Other cognitive constraints	**2 (3.6)**	**1 (1.8)**	**1 (1.8)**
Therapeutic jurisprudence	**2 (3.6)**	**3 (5.6)**	**1 (1.8)**
Total	**7 (12.5)**	**7 (12.5)**	**2 (3.6)**

Cells in bold represent areas of research where factors related to emergency response decision-making were lower than expected, if all narratives were evenly distributed across macro-, meso-, and micro- levels.

## Results

It is necessary to capture and present the distribution of research to help identify any gaps within the evidence-base and frame any narratives toward the distribution of research, and the potential sparsity of research. In order to achieve this, a bibliometric analysis was first undertaken to capture the (i) distribution of research and sparsity of research, and (ii) to frame the primary and secondary narratives by their macro-, meso-, and micro- characteristics. The subsequent holistic narrative analysis attempts to deconstruct the pre-existing theory and reconstruct the narrative to allow for the broad interpretations across all levels (e.g., macro-, meso-, and micro- characteristics). By adopting a more holistic approach in the narrative analysis, the emerging themes can be viewed interpedently across all levels, whilst simultaneously capturing the complex interactions between macro-, meso-, and micro- characteristics.

### Bibliometric analysis

**Figure 3:**
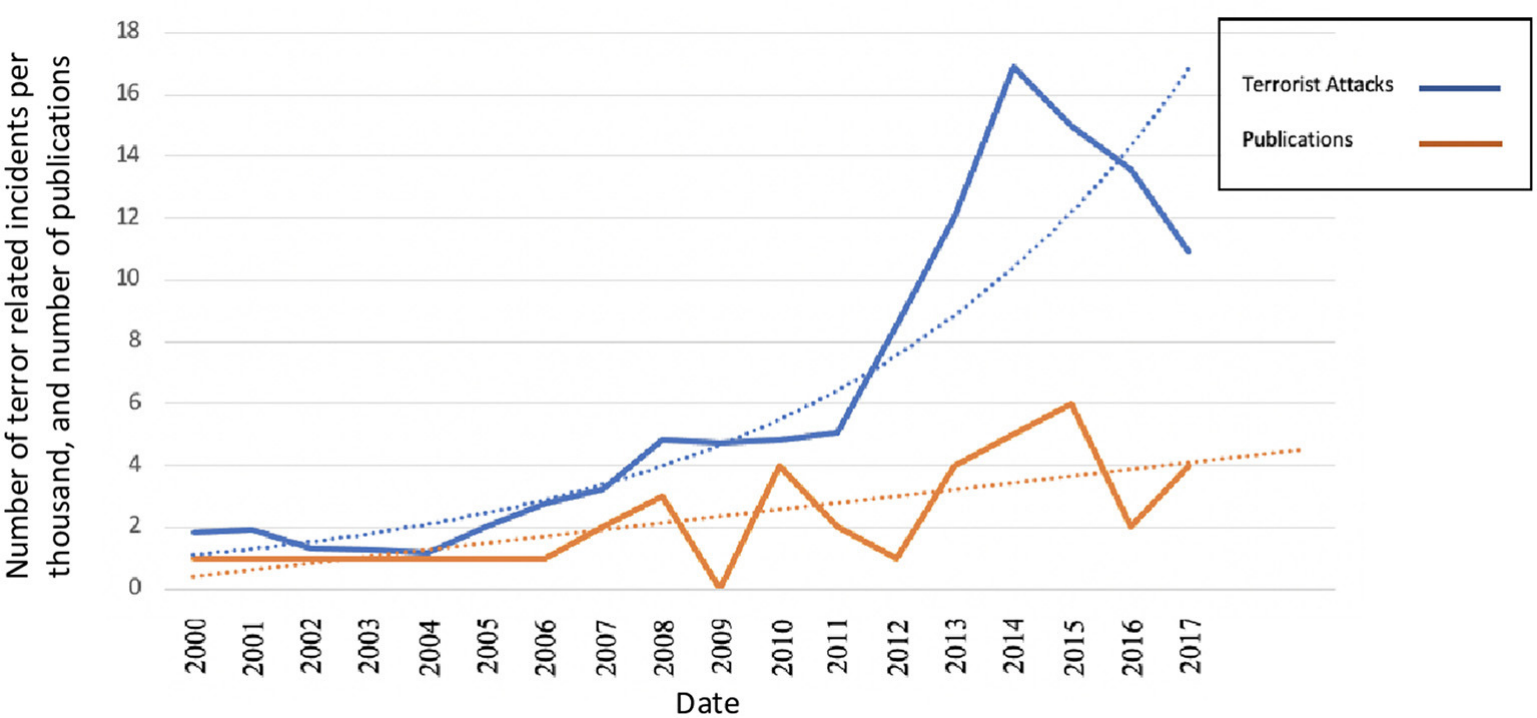
A forecast plot, showing the total number of global terror related incidents (per thousand) and number of published studies between 2000 and 2017. The forecast suggests that as terror related incidents grow exponentially, the number of published studies increase by a factor of 20%. This is not proportionate to the prevalence of critical incidents and suggests that there is a need for greater emphasis on research.

In total, less than 1 (0.02%, *N* = 56) of the studies identified during the initial systematic identification of relevant literature were considered eligible for analysis. The eligible studies represented a majority UK perspectives (58.9%; *N* = 33), with 28.5% (*N* = 16) representing perspectives from the USA, and 12.5% of studies (*N* = 7) having an undefined country of origin. Analysis of publication count revealed an increase in publications over the last 21 years. However, by year of publication, 2018 to 2020 collectively accounted for 33.9% (*N* = 19) of all studies published, with the remaining years accounting for 76.1% (*N* = 37; *M* = 2.1 studies per year) of all studies published. An increase in critical incident events—specifically terrorism appeared to have accounted for 57% of the variance in the increase in publication count between 2000 and 2017. Further, analysis of open access data relating to terror-related incidents (obtained *via* the Global Terrorism Database^1^) revealed a significant correlation between critical incident events and publication count, *r*_s_ (16) = 0.755, *p* < 0.001 (see Figure 3, for a visualization and forecast plot).

Across the 56 studies, 49 unique first authors were identified, with only 7 authors publishing more than one study (Alison, Brown, Cohen-Hatton, Kapacu, Power, van den Heuvel, and Waring). Cohen-Hatton and van den Heuvel were the only authors to examine singular emergency response agencies (fire and rescue services, and police services, respectively), whilst the remaining authors publishing more than 1 study examined decision-making across multi-agency systems.

To assess the heterogeneity of evidence, and overall distribution of evidence across macro-, meso-, and micro- levels, literature was coded to identify first order and sub-level narratives. The cells in Table 3, represent a count of the papers by category with emphasis on the primary, secondary and tertiary narratives. Note, papers often featured more than one narrative. However, this coding did not attempt to capture or code peripheral narrative undertones (i.e., a sub-surface narrative, such as a socio-political factor, that may have existed within the research).

Inspection of the first order narratives revealed moderate heterogeneity, with 48.2% (*N* = 27) of papers aligning with the definition of macro-centric research, 39.3%, (*N* = 22 aligning with the definition of meso-centric research, and 12.5% (*N* = 7) aligning with the definition of micro-centric research. Coding of the secondary narratives revealed that meso-centric and micro-centric research was the most coded categorization (12.3%; *N* = 7), followed by macro-centric (7.1%; *N* = 4). Thirty-eight papers did not have an identifiable secondary narrative. Coding at the tertiary level, revealed that 1.8% (*N* = 1) of the studies had macro-centric characteristics, with micro-centric characteristics accounting for 3.6% (*N* = 2).

At a more fine-grain level, interoperability, communication, and coordination were the most identified factors (28.6%; *N* = 16), followed by political, policy, and procedural factors (23.2%; *N* = 13). A full breakdown can be found in Table 3.

### Holistic narrative analysis and discussion

Six narratives that were thought to comprise decision-making for critical incident response emerged from the analysis: (1) political reform and modernization of emergency management doctrine; (2) difficulties of operating under austerity; (3) uncertainty and accountability; (4) inter-intra government and organizational ethics; (5) failures in collaborative information networks; and (6) limited research-focused horizon scanning.

Each narrative was considered at the primary and sub-level and included consideration for where states-of-emergencies revolve around a *state of preparing* and *responding*. For example, macro-centric analysis identified factors that were influenced by the interactions between geo-political, legal and ethics dimensions; meso-centric concerned the identification of communication networks and the stratification of organizations; and micro-centric analysis identified localized cognitive-driven factors that impacted decision-making (e.g., decision inertia). However, there remains a complex interaction between the characteristics of each factor. For instance, the macro-level factor of political reform and micro-level factor of least-worst decision-making were found to intersect, creating an interdependent system of challenges. The subsequent narratives, therefore, attempt to capture this.

#### Narrative one: Political reform and modernization of emergency management doctrine

Political reform and modernization were found to be integral in enhancing emergency response resilience and preparedness to emerging and increasing threats (Kapucu, 2009). This was also identified in several papers that discussed the need for political, policy and procedural reform (e.g., May, 2004; Banipal, 2006; Gerber, 2007; Scavo et al., 2008; Kapucu, 2009; Birkland and DeYoung, 2011; Kapucu and Garayev, 2011; Stevens, 2020). For example, a 2001 review of emergency response and preparedness procedures highlighted the complexity and lack of capacity for information provision, action, and management of emergency response at local levels (Home Office, 2001). The review highlighted limited centralized focus, leading to politically driven reformation [e.g., when the UK adjusted its emergency management systems and frameworks to a centralized governing body—the Civil Contingencies Secretariat (CCS)]. However, legislative reforms of emergency management structures and frameworks are not uncommon (see Table 4, for a full breakdown of the evolution of key legislative reforms).

**Table 4:** Key legislative reforms and reviews of emergency management structure and frameworks.

**Key legislation and reviews**
The Civil Defense Act 1948
The Local Government Act 1972, Section 138
The Control of Industrial Major Accidents Hazard (CIMAH) Regulations 1984
The Civil Protection in Peacetime Act 1986
The Local Government and Housing Act 1989
The 1989, 1991, and 1997 peacetime emergency planning reviews
The Civil Defense (general local authority functions) Regulations 1993
The 2001 to 2002 emergency review
The Civil Contingencies Act 2004

Politically driven reforms have been found to be associated with centralizing a framework for responding and preparing for emergencies at national, regional, and local levels (O'Brien and Read, 2005). Further, such reformation supports resilience through identifying potential challenges, assessing, and managing contingencies, and planning for future risk (Civil Contingencies Secretariat, 2009). However, it is postulated that politically driven fiscal measures (e.g., austerity) undermine centralized frameworks, reducing resilience and increasing cognitive vulnerabilities (e.g., Wright, 2016). Policy that has attempted to direct and govern under-resourced multi-agency response have been identified as not *fit for purpose* (House of Commons, 2020; Laufs and Waseem, 2020). For instance, Stevens (2020) highlighted the potential risk of policy-related bias when scientific evidence entered politically driven policy development (e.g., COVID-19). In other words, ministers often trawl through evidence to suit a specific agenda (e.g., see Stevens, 2007), manipulating scientific guidance, rather than following the scientific evidence (Stevens, 2020).

The Civil Contingencies Secretariat (CCS) also hold responsibility for bringing together those lessons learnt from previous emergency response outcomes, at all levels, whereby a multi-agency response was needed. Whilst it was found that these well-established information networks informed cross-governmental capabilities (e.g., Chen et al., 2008; Gil-Garcia et al., 2016; Waring et al., 2018), they seldom delivered operational emergency response change in practice to prevent repetition of faulty decisions (Pollock, 2013). This realization appears to be in direct conflict with the core purpose of the CCS, as the lessons learnt only serve to remind multi-agency systems and individual response agencies of their potentially transgressive actions. In other words, lesson learned do not necessarily translate to effective future decision-making, as decisions are often found to be made in context to an incident (Rebera and Rafalowski, 2014), as agencies focus toward least-worst decisions, rather than optimal outcomes (Alison et al., 2018).

The reformation of centralized governing bodies highlights insufficiencies of UK government emergency response management structures (Kapucu, 2009). For example, the Civil Contingencies Act (Civil Contingency Act, 2004) introduced singular legislative and operational frameworks (e.g., JESIP) that provide emergency response decision-makers with civil protection legislation. This enables key decision-makers to define an emergency; identify clear boundaries, responsibilities, and roles for multi-agency systems; allows senior decision-makers to explore duties of local and governmental agencies; and offers governmental bodies legislative powers (O'Brien and Read, 2005). However, it was found that the CCA was not always considered by political and policy decision-makers (House of Commons, 2020; Stevens, 2020). For instance, in response to the COVID-19 pandemic, it was identified that the diversity in policing roles and responsibilities given to operational policing teams hindered effective decision-making. It was believed that operational priorities and duties often evolved beyond the operational capabilities of policing teams. Policing teams were required to enforce restrictive community measures using militarized approaches, which reduced the overall professional standards of community-focus and collaborative policing (Laufs and Waseem, 2020). Further, government legislation, which purports to minimize operational harms and enhance decision-making (e.g., Coronavirus Act, 2020) potentially hinders emergency response decision-making, as there has been minimal public and parliamentary scrutiny (Laufs and Waseem, 2020). Notwithstanding this, there is also little guidance and support to effectively respond and resolve complex taskings (e.g., Stanier and Nunan, 2021; Ghaemmaghami et al., 2021; Newiss et al., 2021), hindering effective decision-making capabilities, as the operational landscapes remains uncertain. Whilst it was recommended in the CCA that emergency response organizations amend their strategic response styles (e.g., Bonkiewicz and Ruback, 2012), it is unclear to what extent multi-agency systems and emergency response personnel can adapt their style of response. It is well cited that multi-agency systems structure their response style toward intra-inter agency collaboration to enhance decision-making (Alison et al., 2013; Laufs and Waseem, 2020). However, whilst there is extensive research that demonstrates the need to improve training and interoperability-related skills (Kapucu and Garayev, 2011; Cohen-Hatton and Honey, 2015; Power and Alison, 2017; Waring et al., 2018; Wilkinson et al., 2019; Brown et al., 2021; Hine and Bragias, 2021), there is limited research that identifies how under-resourced response teams effectively make collaborative decisions. In particular, how collaborative decisions are made in context to leadership and role-taking, and plan enactment in the preparation and response to critical incident events (see, Steigenberger, 2016; for a detail review of case studies and research agendas for multi-agency disaster response).

Ambiguity in what constitutes a critical incident under the CCA (see, Joint Decision Model, 2022, for a detailed definition) is identified as a factor that may limit decision-making. For instance, key decision-makers in the strategic and tactical coordinating groups are not always able to identify critical incidents. It is thought that communication and coordination between emergency response structures lack prior knowledge of CCA legislation and procedures (e.g., JESIP) that assist responding agencies in identifying a critical incident under the CCA (Power and Alison, 2017; Waring et al., 2020). Worryingly, such challenges in identifying a critical incident in the early stages have been found to impede emergency response decision-making (Waring et al., 2020), as decision-makers are unable to rapidly deploy appropriate multi-agency partners from Category 1 (e.g., Police, Fire, Ambulance, etc.) and Category 2 response agencies (e.g., Health and Safety Executive, Department for Transport, Utility Companies, etc; see, Cabinet Office, 2013 for more details).

#### Narrative two: Difficulties of operating under austerity

Over the last decade, fiscal austerity has forced governments to cut response agency budgets, resulting in increased operational pressures, reduced capacity for response, decrease in Category 1 resources, and increased cognitive and macro cognitive stresses (Wright, 2016; Power and Alison, 2017). In other words, austerity has been found to impede effective decision-making. For instance, response commanders are often required to resolve incidents quickly, rather than effectively, to free up resources (Power and Alison, 2017). Moreover, commanders are more likely to triage their decision based on responder wellbeing and capability (e.g., Power and Alison, 2017), as burnout prevalence within the emergency services has become more prevalent with exposure to long-term response efforts (e.g., long-term exposure to response efforts in wildfires; Miller and Mach, 2021). It seems apparent then, that decision-making under these pressures potentially derails operational priorities, as organizational priorities shift toward self-preservation and therapeutic jurisprudence (e.g., Rutkow et al., 2011; Smith and Milne, 2018). Larkin and Arnold (2003) highlighted the challenge of resource allocation, as decisions are triaged based on resource allocation. Operating under these contexts has implications for effective response management, as austerity has been widely reported to undermine response resilience, contributing to increased political, organizational, and individual vulnerability for current and future threats (e.g., Wright, 2016). However, whilst austerity measures have consequences for emergency response decision-making, it is important to highlight that there is a general lack of research that explores the extent to which austerity has comprised effective response decision-making.

#### Narrative three: Uncertainty and accountability

The National Security Strategy (NSS) sets out how multi-agency systems address and manage diverse challenges to security-related critical incidents (e.g., terrorism; HM Government, 2015). The NSS also guides senior decision-makers assessments of security threats, as the threats faced by the UK increase in “*scale, diversity and complexity”* (HM Government, 2015; p. 15). However, whilst the aim and purpose of this strategy has provided direct guidance to strategic, and has driven UK security priorities (e.g., enhancing local and national security capabilities), these strategies do not always mitigate against faulty decision-making (Alison et al., 2018). This includes acknowledging factors of accountability, uncertainty, temporality, and cultural and historical concepts (Alison et al., 2018). For instance, the NSS and the national security risk assessment (NSRA) lack principles of accountability—a construct that refers to the expectation that an individual or organization may be evaluated by a salient audience (e.g., CCS). In part, this may be because operational response agencies and frontline responders favor self-preservation (Alison et al., 2018). Further, anticipating future accountability is thought to impede situational assessments (Alison et al., 2013), limit pro-social behaviors (Frink and Klimoski, 2004), limit effective plan execution in aid of common strategic and operational goals (Gollwitzer and Moskowitz, 1996), and limit the ability to discern relevant versus irrelevant information (Waring et al., 2018).

High levels of uncertainty—a crucial component in governing strategic decision-making (van den Heuvel et al., 2012b)—have also been found to potentially influence decision-making, as multi-agency systems address higher levels of uncertainty through risk avoidance strategies (van den Heuvel et al., 2012b). Where future threats are uncertain, diverse in nature, and complex, multi-agency systems deploy risk avoidance strategies to avoid actions that might lead to potentially negative consequences (e.g., decision inertia; Alison et al., 2015a,b; Shortland et al., 2020). For example, when perceptions of uncertainty are high, response personnel are more likely to narrow their focus toward negative consequences, and thus become more risk avoidant to preserve self-serving issues, in lieu of community protection; defer choice; or favor an act of omissions over commission (van den Heuvel et al., 2012a,b). In summary, the results suggest that the characteristics of critical incident events often exacerbate perceptions of uncertainty. As emergency response organizations adopt a more risk adverse strategy to help promote self-serving bias, and reduce the negative consequences associated to accountability (i.e., future inquiries into actioned decisions) current decision-making models are not considered to be robust. In other words, the prescriptive nature of current decision-models appears to promote triaging systems that focus on community confidence and not community protection.

#### Narrative four: Inter-intra governmental and organizational ethics

Ethics are crucial for social, political, and economic practices, particularly when framed in the context of emergency response (e.g., French and Raymond, 2009; Rutkow et al., 2011; Kalajtzidis, 2016; Leider et al., 2017). When multi-agency systems become stretched beyond their operational capabilities, allocation of resources, civil protection response strategies, and duties to vulnerable persons become an ethically and legally complex problem (e.g., Leider et al., 2017; Gostin et al., 2020). However, in practice decisions are often determined by the best possible, or least-worst outcome (Alison et al., 2018), utilizing effective information sharing networks (Waring et al., 2018) and triaging processes (e.g., WISCI; Smith and Milne, 2018) rather than focusing specifically on ethical and legal guidelines. Ethical guidance is also an increasingly common component of emergency management frameworks (e.g., see Leider et al., 2017), as triaging systems rely on technological solution to inform operational, tactical, and strategic decision-making (e.g., the ethical use of Big Data through social media and mobile data mining; French and Raymond, 2009; Crawford and Finn, 2015). However, ethical principles are considered too ambiguous, and present potential operational and strategic risk (Crawford and Finn, 2015) as frontline responders and response agencies struggle to fully comprehend and apply ethical practice. That is, responders focus toward triaging *why* ethics was needed, rather than understanding the practical application and implementation of ethical principles (Leider et al., 2017).

Integrating ethical principles requires a collaborative and coordination multisystem command structure (Jennings et al., 2016). Further, strong decision-making frameworks are necessary to instill confidence and promote inter-intra-agency communication and coordination through common goals for preparedness planning, emergency response, and post-event recovery (Waring et al., 2018). However, it is suggested that multi-agency systems seldom achieve synergetic coordinated collaboration (Chen et al., 2008; House et al., 2014; Alison et al., 2015b; Waring et al., 2018) as operational end goals consistently differ between response organizations, and organizational structures.

#### Narrative five: Failures in collaborative information networks

Public inquiries into critical incident events have repeatedly highlighted a lack of interoperability as a key factor in response outcomes (Waring et al., 2018; Hine and Bragias, 2021). However, the extent to which multi-agency systems facilitate effective interoperability remains an area of key interest. Whilst it is widely agreed that interoperability enhances critical incident response (e.g., Chen et al., 2008; House et al., 2014; Waring et al., 2018; Hine and Bragias, 2021) it is largely dependent on an organization's ability to develop a shared characterization of a critical incident event (e.g., do responding agencies have a shared operational picture; Salas and Cannon-Bowers, 2001). For example, whether responding agencies have sufficient capability to share information, both reliably and quickly (e.g., information networking technologies; Allen et al., 2014; Waring et al., 2018). Further, whether agencies have the desire to collaborate between other responding agencies (e.g., Chen et al., 2008; Kapucu and Garayev, 2011; Alison et al., 2015b).

Kapucu and Garayev (2011) highlighted that whilst decision-making performance was relatively satisfactory in response to Hurricanes Katrina and Rita in 2005, there were failures in interoperability networks, as communication network capabilities were not fit for purpose; information was not always disseminated due to a lack of trust between agencies; and inter-agency values differed. In addition, the phase one report into the 2019 Grenfell Disaster highlighted several challenges associated with interoperability, as response agencies were not able to fully develop a shared situational awareness. In part, this was due to communication technologies not being fit for purpose, responding agencies were not able to effectively utilize specialist communication technologies, and internal communication policies were not fully understood in context of the on-going events.

In line with current recommendations, agencies can facilitate interoperable decision-making through system monitoring (see, Healey et al., 2009). However, despite facilitating technologies purportedly aiming to enhance decision-making capabilities (House et al., 2014), developing interoperability remains a challenge, as responding agencies seldom have the operational experience of working collaboratively in extreme contexts (Waring et al., 2018; Brown et al., 2021). In addition, standard operating procedures, potentially hinder effective decision-making. This is because the response to a critical incident event often results in organizations adopting a consequentialist approach—where agencies undertake rapidly evaluating courses of action (i.e., on the spot decision-making; Rebera and Rafalowski, 2014) without consideration of the wider operational objectives and operating procedures (see, Shortland et al., 2020). Standard operating procedures were also found to assume an operational hierarchy, whereby commanding personnel (e.g., Gold Command) were assumed to know best how objectives should be achieved. As such, there is a general expectation amongst responding agencies that command strategies and tactics should be followed (e.g., Critical Incident Management Guidance; Home Office, 2021). Whilst hierarchal structures within response agencies reflect a centralized strategic and tactical decision-point, where command personnel operate a “top-down” approach to decision-making, hierarchical structures in emergency response can be counterproductive. In practice, this is due to a lack of consistency in communication channels across teams (e.g., SSG and TSG). This impedes fluid communication (Brown et al., 2021), as decision priorities vary across responding agencies, limiting shared operational understanding and plan formulation (House et al., 2014; Waring et al., 2018) as decision-makers fear breaching operational norms (e.g., Robert and Lajtha, 2002).

A lack of coordination between response agencies (Smith and Dowell, 2000), multi-agency authorities and conflicts of interest (Chen et al., 2008) have been widely discussed as potential factors that limit effective interoperability. In part, this is due to inter-and-intra agency information communication networks disseminating incomplete information that aids decision-making (e.g., Banipal, 2006). For example, Salmon et al. (2011) found that multi-agency communication networks disseminate incomplete information between agencies, resulting in a misunderstanding of the information communicated, limiting interpretability of the information, and consequently resulting in poor information management. Further, a lack of understanding and trust between agencies limit awareness of operational capabilities. This often results in agencies developing independent operational priorities and failing to formulate common operational end-goals (Salmon et al., 2011). Whilst a multi-agency response is required to mitigate least-worst outcomes, the lack of trust, understanding, and failure in communication networks and strategies only serve to exacerbate response challenges.

#### Narrative six: Future research-focused horizon scanning

Experimentally examining all the factors, at a holistic level (e.g., Macro-, Meso, and Micro- level), is crucial to advancing decision-making (Brown et al., 2020). Yet, research remains in its infancy, as decision-making challenges have not yet been captured through empirical formats (Power, 2018; Brown et al., 2020). For example, using technological innovation to empirically examine critical incident decision-making is still a developing area of scientific understanding (Eyre et al., 2012; Alison et al., 2013; Crego and Harris, 2017; Brown et al., 2020). Brown et al. (2020), whilst supportive of innovation and technology, suggests that high-fidelity simulation research lacks context validity, limiting the translation of theory to practice. Further, Bayesian statistical approaches (i.e., an approach to assess epistemological uncertainty in fields of study where there is a lack of knowledge about a fundamental phenomenon) seldom feature in research. For example, Brown et al. (2020) articulated the necessity to utilize more robust and powerful statistical approaches to incorporate prior expectation, without the need to assess critical incident data independently for each study [see, Zyphur and Oswald (2015); for a more complete understanding of Bayesian approaches]. Whilst this is not a suggestion that research cannot provide crucial insights, it does suggest that the validity and impact of current research is limited.

## General discussion

A synthesis and holistic narrative analysis of emergency response research, examined under a macro-, meso-, and micro- lens, identified several key narratives associated with decision-making. Understanding decision-making challenges are seen as crucial to enhancing critical incident decision-making. For example, factors, such as interoperability (Waring et al., 2018; Shortland et al., 2020) were widely found to enhance communication and response coordination, and communication fluidity in respect of multi-agency networks (e.g., Brown et al., 2021). Further, the current study found that research should seek opportunities to better understand challenges *in-situ* of critical incident events and seek innovative solutions to best mitigate challenges to decision-making (i.e., policy and procedural reform, simulation training, and reconsideration of ethical compliance (e.g., Crawford and Finn, 2015; Brown et al., 2020).

Additionally, an examination toward the dispersion and heterogeneity of the evidence suggested that whilst research has increased in recent years, research has primarily focused toward macro- and meso- centric characteristics. For example, it appeared that meso-level issues (e.g., interoperability, communication, and coordination of multi-agency response teams) were of primary interest, with micro-level characteristics identified as receiving relatively little attention. However, it is acknowledged that the wider literature offers deeper insights into the potential micro-level issues (e.g., cognitive issues; Prike et al., 2022). However, it is recommended that these issues should attempt to reframe decision-making in context of critical incident events. In addition, in light of advancement in technology there should be greater emphasis given to empirical assessment (Brown et al., 2020). For example, high fidelity simulation technologies have, in recent years, offered new methodological solutions to decision-making research whilst simultaneously maintaining realistic psychological response.

In terms of quality of evidence, a majority of the papers selected for inclusion were deemed as middling or low quality, as the eligible articles seldom adopted scientifically rigorous experimental epistemology. This is unsurprising, as research has seldom taken opportunities to experimentally assess decision-making, preferring to seek first-hand narrative experience into the unique challenges associated with critical incidents (Brown et al., 2020). In part, this might be explained by the lack of methods suitable for experimentation. For instance, experimentally rigorous research that exploits high-fidelity simulation design rarely considers whether research has psychological and physical fidelity (Kozlowski and DeShon, 2004; Hochmitz and Yuviler-Gavish, 2011). There is also no assessment as to whether response agencies perceive simulation design as representative of a sophisticated life-like environment (i.e., did the environment mimic or reproduce human physiological and psychological affect?). Further, variation in the configuration of simulation-based technologies and methods lack comparable context: as of yet, there is no comparable contextual application of simulation-based technologies [e.g., should simulation methods be used for educational purposes, or for research purposes? (Jensen and Konradsen, 2018)] that has received direct empirical assessment. For instance, Jung (2022) undertook research to assess the applicability for virtual reality simulation (VRS) in disaster response preparedness. Whilst there was some suggestion that VRS could be used as a cost-effective adjunct to current training programmes, there remains several challenges when considering the integration of simulation technologies in training and research. Firstly, simulation technologies require sophisticated computer modeling and environmental depiction (see, WUI-NITY; Wahlqvist et al., 2021). Currently granularity and computational performance limits simulation fidelity and validity. Further, simulation technologies must model a priori and a posteriori of actual incidents to validate simulation models and assess user effect (i.e., to assess a simulation default setting). There are on-going efforts to improve simulation technologies, however, the variability and uncertainty in critical incident response limit any stochastic results.

Research should seek opportunity to experimentally examine decision-making challenges in context of critical incidents. Given advancements in technological innovations, it is now possible to expose multi-agency systems and responders to simulated critical incident events and observe their decision-making processes *in-situ* (Bell et al., 2018). This is not to say that prior research is not credible nor noteworthy—such research underscores the importance of decision-making of those who have direct experience of critical incident events. However, it does provide precedent for future research, and enables research to draw upon pre-existing knowledge for future analysis (e.g., Bayesian statistical approaches). Whilst the papers reviewed in this study often examined macro- and meso-centric factors, the varying narrative dimensions demonstrate that research does have a moderate level of heterogeneity. This is important, as it provides some suggestion that research can be examined more holistically, rather than in isolation. In other words, future research should consider examining heterogeneous factors, moving away from homogenous perspectives of decision-making.

To continue building this evidence base, future work should seek to identify, examine, and evaluate decision-making challenges in context to critical incident events (e.g., Bell et al., 2018). This would allow for a much deeper and more nuanced understanding of the factors that influence effective critical incident response and offer evidence-informed solutions to mitigate against challenges. This includes, assessing the efficacy of technological innovation (e.g., simulation) and exploiting such innovations in research and training to best enhance operational, tactical, and strategic level decision-making. In addition, a renewed research focus toward understanding micro-centric factors appears logical. It is well established that micro-centric factors (e.g., cognition) influences effective decision-making, but it remains an under-researched area (e.g., to what extent do micro-centric challenges transfer to dynamic, complex, and uncertain events?). Methodically robust validation studies of technological innovations will also contribute to the strength of evidence. This includes improving, validating, and modeling complex critical incident scenarios for training and research purposes. Presently, there is limited evidence to methodologically validate the use of simulation technologies in context of critical incident events. Future research may wish to extend this evidence and offer technologically driven solutions to a much-needed field of research.

## Data availability statement

The original contributions presented in the study are included in the article/supplementary material, further inquiries can be directed to the corresponding author.

## Author contributions

BM, RM, AS, and AM: theory conceptualization and formal analysis. BM, RM, AS, ER, and GD: methodology and analysis. BM: data collection and synthesis and writing—draft preparation. BM and ER: inter-rater reliability assessment. BM, RM, AS, AM, and GD: writing—review and editing. All authors have read and agreed to the publication of this manuscript.

## Footnotes

^1^https://www.start.umd.edu/gtd/. For full access to the data, see https://ourworldindata.org/terrorism.
